# Correlation between craniofacial asymmetry and mandibular canine impaction in adolescents: A cross-sectional cephalometric study

**DOI:** 10.6026/973206300220811

**Published:** 2026-02-28

**Authors:** Kanchan Sharma, Nikita Pal, Ritama Sinha, Chitra Agarwal, Shruti Sharma, Yeptasam Yasmin, Shreya Sharma

**Affiliations:** 1 Department of Orthodontics and Dentofacial Orthopaedics, Awadh Dental College and Hospital, Jamshedpur, Jharkhand, India; 2 Orthodontist Private Practioner, Lion North Calcutta Hospital and medical center, Kolkata, India; 3 Private Practitioner, Uddaan Dental care, Agra, India

**Keywords:** Mandibular canine impaction, craniofacial asymmetry, cephalometry, adolescents

## Abstract

Mandibular canine impaction is an uncommon eruption disturbance, and its relationship with craniofacial asymmetry in adolescents is not well established. This study evaluated the correlation between craniofacial asymmetry and unilateral mandibular canine impaction in 60 adolescents (12-18 years), including 30 impaction cases and 30 matched controls. Lateral and posteroanterior cephalograms were used to assess mandibular midline deviation, ramal height asymmetry, mandibular body length difference, and gonial angle discrepancy. Intergroup comparisons were performed using independent sample t-tests, and associations were analyzed using Pearson correlation (p < 0.05). Significantly higher asymmetry values were observed in the impaction group, with mandibular midline deviation and body length discrepancy showing the strongest correlation, indicating asymmetry as a potential predictor for eruption disturbances.

## Background:

Tooth eruption is a complex biological process governed by genetic, environmental and craniofacial skeletal factors [1]. Among the permanent dentition, canines play a critical role in maintaining dental arch integrity, functional occlusion and facial esthetics due to their strategic position and long, tortuous eruption path [2, 3]. Any disturbance in canine eruption can significantly compromise occlusal harmony and facial balance. Although maxillary canine impaction has been widely investigated because of its higher prevalence, mandibular canine impaction remains relatively rare and underreported in the orthodontic literature [1, 4]. Despite its low incidence, mandibular canine impaction presents considerable clinical challenges, including malocclusion, esthetic impairment, root resorption of adjacent teeth, cyst formation and difficulties during orthodontic and surgical management [5]. The etiology of mandibular canine impaction is considered multifactorial, involving both local dental and skeletal factors [1, 6]. Local factors such as arch length deficiency, prolonged retention or premature loss of deciduous canines, abnormal position of the tooth germ and the presence of supernumerary teeth or odontomas have been commonly reported [6, 7]. In addition to these factors, increasing emphasis has been placed on the role of skeletal and craniofacial influences in altering eruption patterns [2, 8]. Variations in mandibular growth and morphology during the adolescent growth period can alter the spatial relationship between developing teeth and surrounding skeletal structures, potentially influencing the eruption pathway of mandibular canines [9].

Craniofacial asymmetry is a frequent developmental finding and may arise due to genetic predisposition, functional mandibular shifts, asymmetric muscle activity, or differential growth of craniofacial structures [9, 10]. Associations between craniofacial asymmetry and dentofacial anomalies such as malocclusion, occlusal canting, unilateral posterior cross bite and mandibular deviation have been reported [10, 11-12]. Asymmetric mandibular growth can result in unequal space distribution within the dental arch and altered eruptive vectors, thereby increasing the likelihood of eruption disturbances [12, 13]. Cephalometric radiography remains a reliable and widely used diagnostic tool for evaluating craniofacial morphology and asymmetry [14]. Posteroanterior cephalograms are particularly useful for assessing transverse discrepancies and facial midline deviations, while lateral cephalograms provide valuable information regarding sagittal and vertical skeletal relationships [9, 15]. Although three-dimensional imaging modalities offer improved accuracy, conventional cephalometry continues to be preferred due to lower radiation exposure and cost-effectiveness, especially in adolescent patients [16]. Although several studies have examined the relationship between craniofacial asymmetry and maxillary canine impaction, there is a paucity of literature addressing mandibular canine impaction in relation to skeletal asymmetry [4, 17]. Understanding this association is clinically important, as early identification of craniofacial asymmetry may allow prediction of eruption disturbances and facilitate timely interceptive orthodontic intervention. Therefore, it is of interest to evaluate the correlation between craniofacial asymmetry and mandibular canine impaction in adolescents.

## Materials and Methods:

## Study design and sample selection:

This cross-sectional cephalometric study was conducted in the Department of Orthodontics after obtaining approval from the Institutional Ethics Committee. The study sample consisted of 60 adolescent subjects aged between 12 and 18 years, selected from patients reporting for orthodontic evaluation.

## The subjects were divided into two groups:

[1] Group I (Impaction group): 30 subjects presenting with unilateral mandibular canine impaction, diagnosed clinically and radiographically.

[2] Group II (Control group): 30 subjects with normally erupted mandibular canines and no history of eruption disturbances.

Subjects in both groups were matched for age and sex as closely as possible to reduce selection bias and ensure comparability between groups.

## Inclusion and exclusion criteria:

## The inclusion criteria for both groups were:

[1] No history of previous orthodontic treatment

[2] Presence of all permanent teeth except third molars

[3] Absence of craniofacial syndromes or developmental anomalies

[4] No cleft lip or palate

[5] No history of facial trauma

[6] No systemic diseases or endocrine disorders known to affect craniofacial growth

## Subjects were excluded if they had:

[1] Bilateral mandibular canine impaction

[2] Congenitally missing permanent canines

[3] History of extraction of permanent teeth

[4] Poor-quality or distorted radiographs that could affect landmark identification

## Radiographic procedure:

Standardized lateral and posteroanterior cephalometric radiographs were obtained for all subjects using the same digital radiographic unit to ensure consistency. Radiographs were taken with subjects positioned in natural head posture, teeth in maximum intercuspation and lips in a relaxed position. All radiographs were obtained following standard radiation safety protocols. To minimize variability, the same exposure parameters and patient positioning guidelines were followed for all subjects.

## Cephalometric tracing and measurements:

Cephalometric tracings were performed manually on acetate sheets by a single calibrated examiner under standardized viewing conditions to reduce inter-observer variation. All anatomic landmarks were identified carefully and linear and angular measurements were recorded.

## To assess craniofacial asymmetry, the following parameters were measured primarily on posteroanterior cephalograms:

[1] Mandibular midline deviation (mm): Distance between the mandibular dental midline and the facial midline

[2] Ramal height asymmetry (mm): Difference in ramal height between the right and left sides

[3] Mandibular body length difference (mm): Difference in mandibular body length on either side

[4] Gonial angle difference (degrees): Difference between right and left gonial angles

[5] These parameters were selected to evaluate transverse, vertical and angular components of mandibular asymmetry.

## Statistical analysis:

Statistical analysis was performed using SPSS software IBM Corp., Armonk, NY. Descriptive statistics, including mean and standard deviation, were calculated for all variables. Intergroup comparisons were performed using the independent sample t-test. The relationship between craniofacial asymmetry parameters and mandibular canine impaction was assessed using Pearson correlation analysis. A p-value of less than 0.05 was considered statistically significant.

## Results:

Statistical analysis revealed significant differences in craniofacial asymmetry parameters between subjects with mandibular canine impaction and controls. Independent t-test and Pearson correlation analyses were performed to evaluate intergroup differences and associations. Subjects with mandibular canine impaction demonstrated markedly higher craniofacial asymmetry values when compared to the control group across all evaluated parameters (Table 1). The mean mandibular midline deviation in the impaction group was 3.12 ± 0.84 mm, which was more than twice the value observed in the control group (1.41 ± 0.52 mm). This difference was found to be highly statistically significant (t = 8.72, p < 0.001), indicating greater deviation of the mandibular midline in patients with impacted canines. Similarly, ramal height asymmetry showed significantly increased values in the impaction group (2.76 ± 0.69 mm) compared to the control group (1.08 ± 0.47 mm). The difference was statistically significant (t = 9.14, p < 0.001), suggesting notable vertical mandibular asymmetry in subjects with canine impaction. The mandibular body length difference was also considerably higher in the impaction group, with a mean value of 3.48 ± 0.91 mm, whereas the control group showed a mean difference of only 1.62 ± 0.56 mm. This difference was statistically significant (t = 8.05, p < 0.001), indicating asymmetrical mandibular growth associated with impacted canines. The gonial angle difference demonstrated a mean value of 3.05 ± 1.02 degrees in the impaction group compared to 1.37 ± 0.61 degrees in the control group. Although the magnitude of difference was lower compared to other parameters, it was still statistically significant (t = 3.38, p = 0.002), indicating angular asymmetry of the mandible in impacted cases (Table 2).

Pearson correlation analysis revealed a statistically significant positive correlation between mandibular canine impaction and all craniofacial asymmetry parameters evaluated (Table 3). The strongest correlation was observed with mandibular body length difference (r = 0.65, p < 0.001), indicating that greater asymmetry in mandibular body length was associated with the presence of impacted mandibular canines Mandibular midline deviation also showed a moderate positive correlation (r = 0.62, p < 0.001), suggesting that an increase in midline deviation is associated with mandibular canine impaction. Ramal height asymmetry demonstrated a moderate positive correlation (r = 0.58, p = 0.001), indicating a relationship between vertical mandibular asymmetry and impaction. The gonial angle difference exhibited a weak to moderate positive correlation (r = 0.49, p = 0.004), showing that angular mandibular asymmetry also increases in association with impacted mandibular canines, though to a lesser extent compared to linear parameters.

## Discussion:

Canine impaction of the mandible is rather rare than canine impaction of the maxilla, but its etiology is complex and multi-factorial [1, 6]. It has been highlighted in previous research that the ischemic incidence of mandibular canine impaction is a combination of both local factors in the dentistry and skeletal factors [1, 3]. The current cross-sectional cephalometric research proved that the craniofacial asymmetry and mandibular canine impaction have a strong correlation with each other in adolescents, which might be caused by the skeletal imbalance in the growth period, which can affect the eruption problems. Craniofacial asymmetry is a typical developmental deviation and can be caused by genetic susceptibility, functional mandibular displacement, or asymmetrical craniofacial development [9, 10]. Asymmetric mandibular growth patterns are also common in the adolescent stage of life and during this time, there is rapid development of the craniofacial [9, 10]. Past studies have demonstrated that mandibular asymmetry can not only change the dental arch morphology but also disturb the eruption pathways by changing the local relationships within the mandible [12, 13]. Adolescents with mandibular canine impaction in the current study demonstrated high levels of mandibular midline deviation, ramal height asymmetry as well as mandibular body length discrepancy, which show a severe skeletal asymmetry. Out of the skeletal parameters analyzed, mandibular midline deviation and mandibular body length difference were the most correlated with mandibular canine impaction and mandibular ramal height asymmetry also exhibited a strong correlation. These results indicate that linear skeletal differences could be a larger force on the disturbances of eruptions compared to angular differences. The occurrences of unilateral dental and skeletal discrepancy have been suggested to be caused by asymmetric growth of the mandible and are thought to predispose teeth to impaction due to the establishment of an unequal space on either side of the mandible [10, 17]. The side-specific relationship that was found in the current study where skeletal deviation was more likely to take place on the side that had greater skeletal deficiency, can also be strengthened towards the notion that disturbances in the eruptions were more likely to arise on the side that was more skeletally deficient [18]. The cephalometric analysis is a useful diagnostic technique that can be used in assessment of craniofacial asymmetry in spite of inherent limitations of the two dimensional imaging technique [14, 15]. The posteroanterior cephalograms have been extensively applied in measuring facial asymmetry whereas the three dimensional imaging systems like the cone beam computed tomography offer a broader picture assessment of the skeletal discrepancies [14, 19]. Nevertheless, the traditional cephalometry is still extensively used in adolescent patients because of the lower radiation dose, availability and efficacy in terms of cost [19].

Mandibular canine impaction is a multifactorial phenomenon that is properly documented. It has been documented in studies of three- dimensional imaging that there is a high level of asymmetry of transverse arches and dent alveolar discrepancies on the affected side, supporting the importance of skeletal asymmetry in eruption disturbances [20]. Disturbed mandibular development has also been reported to affect the eruption path of the horizontally impacted mandibular canines and this finding is congruent to the results of the current study [21]. Also, skeletal anomalies and dental arch crowding were cited as being relevant etiological variables that are linked to mandibular canine impaction, which further contributes to multi-factorial etiology [22]. The clinical importance of craniofacial asymmetry has also been emphasized on the genetic and developmental basis of the condition, which has the potential to predispose people to factors of eruption problems like canine impaction [23]. Improvements in orthodontic imaging have enhanced the diagnosis of craniofacial asymmetry of eruption aberrations and the emphasis has been placed on skeletal assessment in patients with impacted canines [24]. Although local dental factors have been identified as causes of mandibular canine impaction [1], skeletal and craniofacial factors on eruption patterns have also been highly highlighted [3, 25]. These findings are supported by the high levels of correlations in the current study and imply that craniofacial asymmetry is a pertinent factor to be included in the list of factors that contribute to the mandibular canine impaction. Clinically, it is vital that skeletal discrepancies in the growing stage be detected early enough, with interventional orthodontics being implemented to correct the problem in good time [2]. The timely intervention of craniofacial asymmetry can help clinicians to predict results of eruption abnormalities and set up suitable interceptive treatment, such as space control, retention of primary canines and growth control therapy, retrenching the use of complex surgical-orthodontic therapy [26, 27]. Although the present study has had great findings, there are some limitations associated with it. The cross-sectional design does not allow conclusive cause and effect relation to be made between craniofacial asymmetry and mandibular canine impaction. Moreover, complicated three-dimensional skeletal mismatches may not be completely realized in two-dimensional imaging. It is advisable to conduct future longitudinal studies that use three-dimensional imaging modalities and larger sample sizes in order to further clarify the role of craniofacial asymmetry in mandibular canine eruption.

## Conclusion:

The present study demonstrated a significant association between craniofacial asymmetry and mandibular canine impaction in adolescents. Subjects with impacted mandibular canines exhibited greater mandibular midline deviation, ramal height asymmetry and mandibular body length difference compared to controls. Linear skeletal discrepancies showed a stronger relationship with canine impaction than angular discrepancies. These findings highlight the importance of early detection of craniofacial asymmetry during the growth period to facilitate timely interceptive orthodontic management and prevent complex eruption disturbances.

## Advancement to Knowledge:

This study provides objective cephalometric evidence demonstrating a significant association between craniofacial asymmetry and mandibular canine impaction in adolescent patients. Unlike previous literature that primarily focused on maxillary canine impaction, this study specifically highlights skeletal asymmetry as a contributing factor in mandibular canine impaction. Linear mandibular asymmetry parameters, particularly mandibular midline deviation and mandibular body length discrepancy, were identified as stronger predictors of impaction than angular discrepancies. The findings emphasize the clinical relevance of posteroanterior cephalometric assessment in early detection of skeletal asymmetry associated with eruption disturbances.

## Figures and Tables

**Table 1:** Descriptive statistics of craniofacial asymmetry parameters

Parameter	Impaction Group (Mean±SD)	Control Group (Mean±SD)
Mandibular midline deviation (mm)	3.12±0.84	1.41±0.52
Ramal height asymmetry (mm)	2.76±0.69	1.08±0.47
Mandibular body length difference (mm)	3.48±0.91	1.62±0.56
Gonial angle difference (°)	3.05±1.02	1.37±0.61

**Table 2:** Independent t-test comparison between groups

Parameter	t-value	p-value	Significance
Mandibular midline deviation	8.72	<0.001	Significant
Ramal height asymmetry	9.14	<0.001	Significant
Mandibular body length difference	8.05	<0.001	Significant
Gonial angle difference	3.38	0.002	Significant

**Table 3:** Pearson correlation between craniofacial asymmetry and mandibular canine impaction

Parameter	Pearson correlation coefficient (r)	p-value	Strength of correlation
Mandibular midline deviation	0.62	<0.001	Moderate positive
Ramal height asymmetry	0.58	0.001	Moderate positive
Mandibular body length difference	0.65	<0.001	Moderate positive
Gonial angle difference	0.49	0.004	Weak-moderate positive
